# Nectar Uptake of a Long-Proboscid *Prosoeca* Fly (Nemestrinidae)—Proboscis Morphology and Flower Shape

**DOI:** 10.3390/insects12040371

**Published:** 2021-04-20

**Authors:** Harald W. Krenn, Florian Karolyi, Peter Lampert, Annalie Melin, Jonathan F. Colville

**Affiliations:** 1 Department of Evolutionary Biology, University of Vienna, 1090 Vienna, Austria; florian.karolyi@univie.ac.at; 2 Department of Botany and Biodiversity Research, University of Vienna, 1030 Vienna, Austria; peter.lampert@univie.ac.at; 3 Compton Herbarium, South African National Biodiversity Institute, Newlands, Cape Town 7735, South Africa; annalie.melin@gmail.com; 4 African Centre for Coastal Palaeoscience, Nelson Mandela Metropolitan University, Port Elizabeth 6000, South Africa; 5 Statistics in Ecology, Environment and Conservation, Department of Statistical Sciences, University of Cape Town, Rondebosch, Cape Town 7701, South Africa; jonathan.colville@gmail.com; 6 Kirstenbosch Research Centre, South African National Biodiversity Institute, Newlands, Cape Town 7735, South Africa

**Keywords:** mouthparts, nectar-feeding, insect-flower interaction, flower morphology, nectar quantity, nectar level calculation

## Abstract

**Simple Summary:**

Flies with a particularly long proboscis are characteristic of flower-visiting insects in the Greater Cape Floristic Region of South Africa. We studied an endemic nemestrinid fly species in a small isolated area of semi-natural vegetation where these insects were the only flower visitors that could drink nectar from the available long-tubed flowers of one plant species. We examined the mouthpart structures that are important for nectar uptake and the length and diameter of the proboscis in comparison with the flower sizes. This local one-to-one interaction between the fly population and its nectar host flower gave the opportunity to quantify the nectar resources available for the nemestrinid flies at the study site. By comparing the offered nectar volumes before and after flower visits, the average meal size could be estimated. Assessments of the nectar levels from measured quantities and flower size allowed us to make predictions of how various proboscis lengths could reach nectar inside floral tubes.

**Abstract:**

Several *Prosoeca* (Nemestinidae) species use a greatly elongated proboscis to drink nectar from long-tubed flowers. We studied morphological adaptations for nectar uptake of *Prosoeca* *marinusi* that were endemic to the Northern Cape of South Africa. Our study site was a small isolated area of semi-natural habitat, where the long-tubed flowers of *Babiana vanzijliae* (Iridaceae) were the only nectar source of *P. marinusi*, and these flies were the only insects with matching proboscis. On average, the proboscis measured 32.63 ± 2.93 mm in length and less than 0.5 mm in diameter. The short labella at the tip are equipped with pseudotracheae that open at the apical margin, indicating that nectar is extracted out of the floral tube with closed labella. To quantify the available nectar resources, measurements of the nectar volume were taken before the flies were active and after observed flower visits. On average, an individual fly took up approximately 1 µL of nectar per flower visit. The measured nectar quantities and the flower geometry allowed estimations of the nectar heights and predictions of necessary proboscis lengths to access nectar in a range of flower tube lengths.

## 1. Introduction

Anthophilous insects have evolved a remarkable diversity of elongated mouthparts to take up concealed nectar from flowers [1,2]. As an energy-rich food source, nectar attracts an enormous diversity of flower-visiting insects, including numerous Diptera [3,4,5] that supplement their daily needs regardless of if they pollinate the visited flowers or not [6]. Depending on the plant species, nectar is an aqueous sugary solution that contains mainly glucose, fructose, and sucrose, and small amounts of amino acids, proteins, and varying levels of trace elements [7,8,9]. The amount of nectar available in a habitat has been shown to be both patchy and highly variable, with nectar volumes ranging from less than 1 µL to several milliliters per flower, depending on plant species and flower morphology [10,11,12,13].

Some taxa of flower-visiting Diptera have evolved particularly long proboscises that are specialized to long tubular flowers [3,14,15,16,17]. One striking example is the long-proboscid fly pollination system of the Greater Cape Floristic Region in southern Africa, where a number of anthophilous brachyceran Diptera (Nemestrinidae and Tabanidae) pollinate approximately 200 specialized plant species from at least 10 angiosperm families [15]. Preferred nectar host plants are characterized by long, narrow, straight, or slightly curved floral tubes, containing nectar volumes up to 13 µL with a sugar concentration between 20 and 32% [15]. In particular, the long-proboscid flies of the family Nemestrinidae contain anthophilous species that provide valuable pollination for a large number of endemic plant species [15,18,19,20,21,22]. Nemestrinid flies from the genera *Moegistorhynchus*, *Stenobasipteron*, and *Prosoeca* show exceptionally long, but geographically highly variable proboscis lengths, which are shaped by coadaptation with local nectar host plants [14,15,22,23,24,25].

The genus *Prosoeca* comprises about 40 described species in South Africa [26,27,28]. *Prosoeca marinusi* Barraclough, 2018, is a local endemic species that is restricted to western parts of the South African Bokkeveld Plateau [27], a region hosting one of the most diverse wildflower areas in the Greater Cape Floristic Region [29]. This long-proboscid anthophilous fly is a characteristic feature of the brief spring flowering season and is an important pollinator of several endemic bulbous plants [27,30]. Studies on the mouthpart morphology of *Prosoeca* revealed a complex proboscis morphology [31]. Further investigations of flower handling behavior indicated that flies with longer proboscises take-up more nectar in a single visit, gaining an advantage over shorter proboscid individuals [25,32]. However, these studies did not investigate proboscis morphology in the context of the functional fit with flower shape and did not quantify the nectar volume ingested by flies per flower visit. The present study examines the nectar extracting abilities of *P. marinusi* from its long-tubed, local nectar host plant *Babiana vanzijliae* L. Bolus, 1925, (Iridaceae). We used a small isolated patch of remnant natural vegetation, where *B. vanzijliae* was the only nectar resource for the population of *P. marinusi*, and this fly was the only nectar-feeding visitor of *B. vanzijliae*.

The aim of this study was (1) to examine the proboscis tip where nectar is extracted, (2) to compare length and diameter of the proboscis with the nectar host flower in order to assess nectar accessibility, and (3) to estimate how much nectar is taken up per flower visit. The characteristics of the study site allowed an estimate of the daily nectar standing crop that fuels the activity of the isolated fly population. Our assessment of nectar levels inside the floral tubes allowed predictions of necessary proboscis lengths and the accessibility of nectar.

## 2. Materials and Methods

### 2.1. Study Site

**Figure 1:**
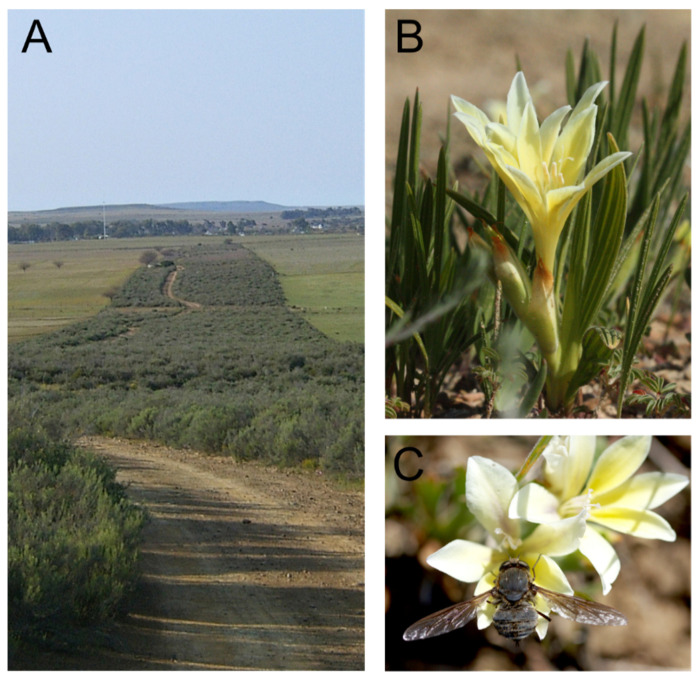
Study area in the Northern Cape Province, South Africa, (31°20′54” S, 19°05′30” E), nectar hostplant and visiting fly. (**A**) A small isolated area with natural Shale Renosterveld vegetation surrounded by transformed areas with grazed farmland. (**B**) *Babiana vanzijliae* (Iridaceae), the only available nectar host plant for (**C**) *Prosoeca marinusi* (Nemestrinidae) at the study site.

The field study was conducted in the surroundings of Nieuwoudtville (Northern Cape Province), a winter-rainfall region in South Africa (742 m a.s.l; 31°20′54” S, 19°05′30” E). The study site was characterized as a near-natural area of 9450 m^2^ with natural vegetation consisting of Nieuwoudtville Shale Renosterveld [33]. The study site was surrounded by farmland used for sheep grazing (Figure 1A). Field observations were performed over three sunny days at the end of August 2017 from 9 a.m. to 3:30 p.m., and the air temperature ranged between 14 °C in the morning and 30 °C in the afternoon. In the study area, only the nemestrinid fly *P. marinusi*, the western honey bee *Apis mellifera*, and several undetermined Meloidae and Hopliini beetles were observed to visit flowers of *B. vanzijliae* during the study period.

### 2.2. Proboscis Morphometry

Details about the distribution and biology of *P. marinusi* can be found in [27]. Flies foraged between 9:55 a.m. to 2:40 p.m. at air temperatures ranging between 17 and 29 °C. Flies (*n* = 80) were caught using hand nets while visiting flowers. Duration of flower-visiting time were observed and measured with a stopwatch. Body and proboscis length measurements were taken in situ from each captured fly using a pair of digital calipers (Helios Digi-Met 1220; maximum accuracy 0.01 mm). Each fly was marked with a unique code on the wing using a permanent marker (Staedtler permanent special Lumcolor F) to prevent measuring recaptured individuals. Mark-recapture trials of 100 individuals were conducted at five other sites over a period of 7 days at the end of August 2017, ranging between 5.7 and 10.5 km from the study area, in order to determine if there was an exchange of individual flies among populations. No exchange of individuals of the isolated populations of *P. marinusi* was detected [34].

To obtain detailed morphological measurements of the proboscis, ethanol (95%) fixed specimens of *P. marinusi* (*n* = 10) were dehydrated, embedded in agar low viscosity resin and sectioned [35]. Sections of 1 μm were cut with a Leica EM UC6 microtome. Semithin sections were stained in a mixture of azure II (1%) and methylene blue (1%) in hydrous borax solution (1%), at 90 °C for 10–20 s. Sections through the proboscises were studied using a Nikon Labophot-2 microscope equipped with a drawing tube; cross section photos were taken using a Nikon Eclipse Ni microscope.

### 2.3. Nectar Host Plant and Nectar Sampling

*Babiana vanzijliae* (sometimes spelled as *B. vanzyliae*) is an endemic Iridaceae of the Bokkeveld Plateau. It grows in rocky sandstone- and tillite-derived soils in fynbos and Renosterveld vegetation [21,36]. This plant grows up to 120 mm high and flowers from August to September. The yellow flowers are slightly zygomorphic with six tepals extending from a long, slender, narrowly funnel-shaped perianth tube (Figure 1B). The lower lateral tepals have whitish median blotches and attach to the upper laterals, forming an oblique lip. The perianth tube contains the stylus that extends from the basal ovary and branches distally over the stamen, and the filaments extend from the upper third of the perianth with the three anthers arranged on one side [36]. Nectar contained approximately 30% sugar (measured with a handheld refractometer) and was secreted at the top of the ovary at the base of the narrow perianth tube where it accumulated inside the tube.

We counted 407 open flowers of *B. vanzijliae* at the study site on one day. In ≈10% of these flowers (*n* = 41), the perianth tube lengths and diameters were measured with calipers (Helios Digi-Met 1220). To estimate the nectar availability in the field, we measured the nectar content of another 52 flowers in the morning before the flies were active (ambient temperature <16 °C) using calibrated glass microcapillaries (P6679 Sigma 5 µL; accuracy 0.1 µL). The nectar was extracted immediately after the flower was cut just above the ovary; a microcapillary was pressed to the bottom end of the cut-off perianth tube. To estimate the amount of nectar taken up by *P. marinusi*, flowers were picked immediately after a fly had finished feeding (*n* = 39) and the remaining nectar was measured. The amount of nectar imbibed was calculated based on the average nectar volume measured before fly activity commenced.

**Figure A1:**
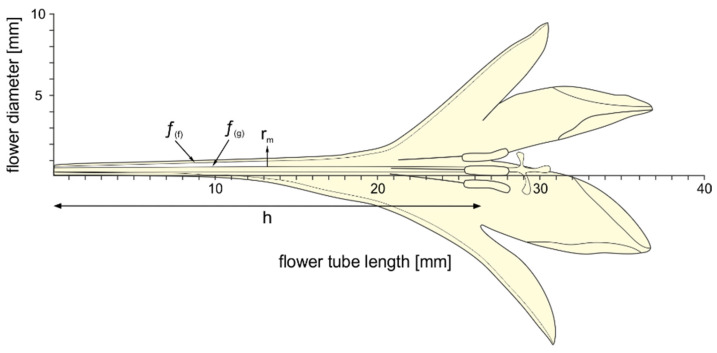
Geometrical model of a *Babiana vanzijliae* flower, perianth tube length (h) measured up to notches between tepals. The linear functions (f) and (g) are defined by the measured mean inner radius at the base of the tube, mean inner radius at mid-length (r_m_), and radius of the stylus (indicated in grey). Rotating the object defined by function (f) around the *x*-axis creates a frustum of a cone using integral calculus (disc method). The subtraction of the volume of the stylus (function (g)) from the total volume (function (f)) allows the determination of the nectar level inside the perianth tube by using the GeoGebra tool (https://www.geogebra.org/m/se2cwx9s, accessed on 16 April 2021).

To calculate the nectar level (nectar height) inside the perianth tube, we measured the length of the perianth tube and the diameter at the base and at mid length and calculated the inner radius by subtracting the tube wall thickness. These values were measured using Image J from cross sections of ethanol preserved flowers using a Nikon Labophot-2 microscope (*n* = 9). The geometry of the perianth tube was estimated as a long and slender frustum of a cone containing a cylindrical stylus (Figure A1).

**Figure A2:**
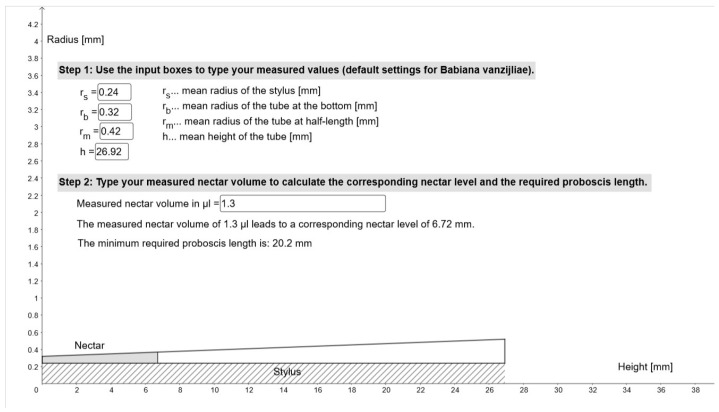
Calculation of nectar height inside a tubular flower (screen shot of GeoGebra); step 1: type in the measured dimensions of the tubular flower; step 2: type in the measured nectar volume (e.g., from micropipette measurements). The tool automatically calculates and illustrates the corresponding nectar level. The tool also provides information on the required proboscis length to reach the nectar. https://www.geogebra.org/m/se2cwx9s, accessed on 16 April 2021.

Based on this geometric assumption, a nectar level calculation tool was developed (using the mathematical software program GeoGebra). This tool allows users to determine the nectar level by inputting the measured values of the flower (inner radii of the tube at the bottom and at half-length; length of the perianth tube; radius of the stylus) in a first step. In a second step, the measured nectar volume (e.g., from micropipette measurements) can be typed in. Based on these inputs, the calculation tool calculates the nectar level, as well as the required proboscis length to reach the nectar (Screenshot Figure A2). We provide the freely available tool and instructions via the link https://www.geogebra.org/m/se2cwx9s (accessed on 16 April 2021). The mathematical backgrounds of the nectar level calculation tool can be found in Appendix A.

The figures were created with CorelDRAW 2018 (Version 20.0.0.633) Corel Corporation, Ottawa, Ontario, Canada and Photoshop CC2018, Adobe, San Jose, California, U.S.A.

## 3. Results

### 3.1. Flower Visiting Behaviour and Proboscis Morphology

*Prosoeca marinusi* visited only *B. vanzijliae* flowers at the study site (Figure 1). Flies hovered over flowers for a short time before the proboscis tip entered the perianth. While the fly lowered down between the tepals (Figure 1C), it inserted its proboscis into the opening of the nectar tube. The total flower visiting time observed was 4 ± 2.1 s (*n* = 21).

**Figure 2:**
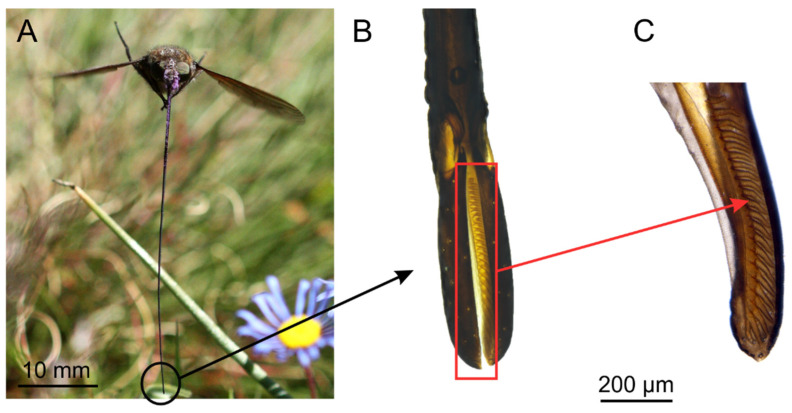
The long-proboscid fly *P. marinusi* (Nemestrinidae) and morphology of the proboscis tip; (**A**) Proboscis in feeding position; (**B**) Proboscis tip formed by pair of labella; (**C**) Median view of labellum shows pseudotrachea canals for nectar uptake through apical end of proboscis.

**Figure 3:**
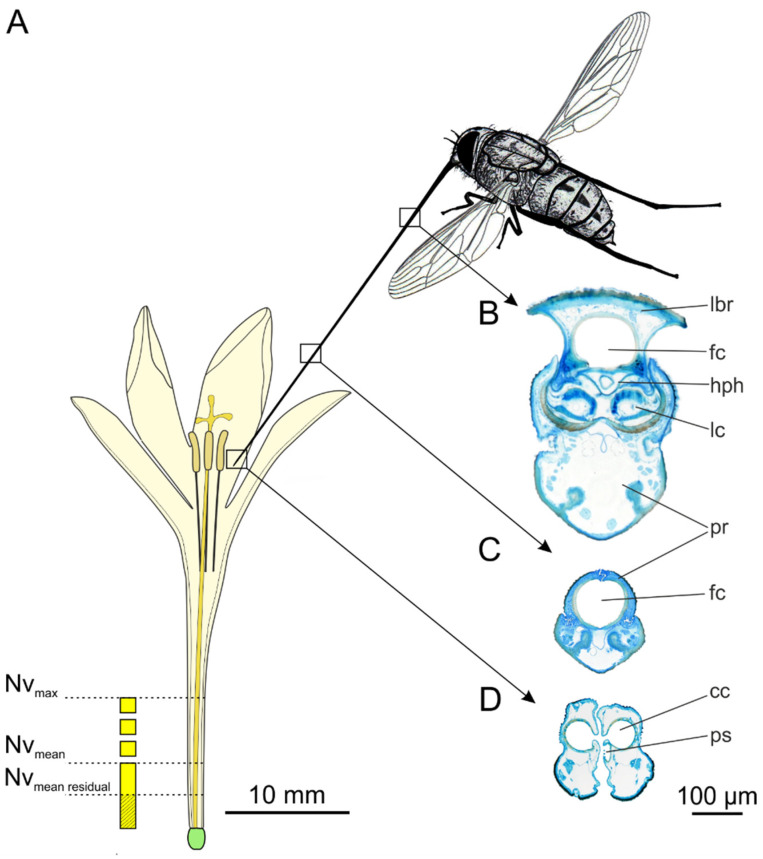
Nectar accessibility for *P. marinusi* from (**A**) an average *B. vanzijliae* flower. Nectar heights calculated using the GeoGebra tool (https://www.geogebra.org/m/se2cwx9s, accessed on 16 April 2021) from measured quantities, modelled with a mean inner diameter and average tube length (*n* = 41). Maximal nectar volume (N_vmax_) resulted in nectar height approximately up to half length. Mean nectar volume before fly activity (N_vmean_, *n* = 44) fills nectar tube up to 20% of tube length. Mean residual nectar volume after fly visited (N_vresidual_, *n* = 39) in the proximal was 10%. (**B**) Cross section of the proximal proboscis. (**C**) Proboscis cross section of distal proboscis that is composed only by the prementum. (**D**) Cross section of the labella at the tip of the proboscis. cc: collecting canal; fc: food canal; hph: hypopharynx; lbr: labrum; lc: lacinia; pr: prementum; ps: pseudotrachea.

Proboscis length was up to almost double the body length and ranged from 23–39 mm (mean 32.68 ± 38.86 mm, *n* = 80) in the studied population (Table 1). The labella formed a short drinking region at the tip of the proboscis (Figure 2) to extract nectar out of the floral tube. The labella measured 0.82 ± 0.04 mm in length, which was equivalent to 2.56% of the proboscis length. The pair of labella had a diameter of 0.22 ± 0.03 mm (*n* = 10) (Table 1, Figure 2B). The median side of each labellum had 20–27 pseudotracheae that extended to the apical margin (Figure 2C). In the proximal third, the proboscis had a rectangular cross section (Table 1, Figure 3B), while the distal proboscis (composed of the prementum only) was nearly round (Figure 3C) with only half the diameter, measuring only 0.21 ± 0.01 mm (*n* = 10) (Table 1). In contrast to the changing shape and composition of the proboscis from the base to the tip, the food canal inside the proboscis remained oval throughout the length (Figure 3B,C). It only slightly decreased in diameter from approximately 0.14 mm proximally to 0.11 mm (*n* = 10) distally. In the drinking region, the labella formed two collecting canals, each measuring 0.07 ± 0.01 mm (*n* = 9) in diameter, where the pseudotracheae entered into the food canal (Figure 3D).

### 3.2. Flower Shape and Nectar Availability

The length of the nectar tubes of *B. vanzijliae* ranged between 19 and 38.25 mm (mean 26.92 ± 5.75 mm, *n* = 41). The perianth was funnel-shaped with a broad opening, which decreased to form a slender tube with a radius of 1.1 mm at the opening. The diameter of the perianth tube tapered down to the ovule where the radius measured 0.32 ± 0.04 mm (Table 2). The volume of the perianth tube was further decreased by the stylus (mean radius 0.25 ± 0.02 mm), which extended from the ovule upwards between the tepals (Figure 3A).

The nectar volumes ranged from 0 (detected in eight flowers) to a maximal amount of 5.3 µL in one flower. On average, we measured 1.30 ± 1.19 µL of nectar per flower (*n* = 52), including empty flowers. In total, the 407 open *B. vanzijliae* flowers contained approximately 530 µL of nectar in the morning before fly foraging activity started. Excluding the flowers without nectar, the mean nectar quantity estimate was 1.54 ± 0.94 µL before the flies were active (Table 3). Taking the different nectar volumes and various diameters of the perianth tubes into account, nectar heights ranged between 0.73 and 10.37 mm (mean 5.12 ± 2.63 mm, *n* = 44) in the population (Table 3). This indicates that the nectar was limited to the lower half of the tube (Figure 3A). A *Prosoeca* fly with an average proboscis length of 32.63 ± 2.93 mm could drink nectar from 42 out of the 44 nectar-containing flowers.

The amount of nectar measured from flowers after a visit from a *Prosoeca* fly ranged from 0 to 2 µL, with a mean quantity of 0.52 ± 0.63 µL (*n* = 39) (Table 3). This indicated mean nectar meal size of 1.02 µL per visit, based on the subtraction of the mean ingested volume from the mean nectar quantity of 1.54 µL per flower.

## 4. Discussion

### 4.1. Functional Morphology of the Proboscis

*Prosoeca marinusi* is a large, agile flying insect with a proboscis that can measure more than double the body length. Its energy demanding hovering flight is fueled by nectar that is taken up by frequently visiting long-tubed flowers. Nectar ingestion through the long food canal is mainly achieved by the action of large suction pumps in the head [32], which is probably aided by the hydrophilic cuticle properties of the food canal and the capillarity of the pseudotracheae of the labella. Capillary forces are expected to facilitate nectar adhesion onto the cuticle structures of the apical drinking region, as seen in muscid Diptera and various Lepidoptera [37,38]. The proboscis of *P. marinusi* is not only very long, but also particularly slender. Although the pair of labella measures less than 0.25 mm in diameter, the proboscis tip only just fits into the similarly narrow basal region of the perianth tube of *B. vanzijliae*. Based on proboscis tip morphology, it can be assumed that the labella are pressed together, indicating that nectar is extracted between the closed labella, as the pseudotracheae open at the apical end. They extend into the longitudinal collecting canal that further opens into the food canal that is connected with the suction pump [31]. Slender labella appear to be a characteristic feature of long-proboscid nectar-feeding Diptera, e.g., Tabanidae [16,39], Acroceridae [40,41], and Bombyliidae [42,43], and is interpreted as an adaptation to long but very thin nectar tubes of host plants. Reich et al. [44] demonstrated that a narrow entrance into the nectar tube is a characteristic feature of flowers that are specialized to pollination by long-proboscid flies in the Greater Cape Floristic Region. In our study, however, the nectar host flower does not show an especially narrow opening, rather, these flowers have a very thin perianth tube proximally where the nectar accumulates and from where nectar is extracted.

Although flowers of *B. vanzijliae* are visited by different insects across its distribution range [21], in this study, *P. marinusi* was the only insect that was equipped with a matching proboscis to reach the nectar. Several studies examine the proboscis lengths in mutualistic long-tongued fly-flower interactions [14,16,23,25,45]. Likewise, the diameter of the proboscis appears to be an important morphological feature that may restrict access to a flower but has not been considered so far. We present data on matching cross-sectional dimensions that are also important for understanding how insects are able to access floral rewards from the narrow nectar tubes of host plants.

### 4.2. Nectar Supply

The nectar volume produced by a single flower can be very small, and foraging insects are forced to visit numerous flowers to meet their daily energy requirements [46]. Several studies have examined the composition and volume of nectar in various plant species in relation to specific pollination syndromes [9,12,47,48,49,50]. However, only a few studies have directly measured the amount of nectar consumed by insects under natural conditions [25] and correlated these data with floral tube depth and proboscis length [51]. Our study site was characterized by a one-to-one interaction between a long-proboscid fly species and its single nectar host plant species. Since no other insects were observed drinking nectar from the long-tubed flowers of *B. vanzijliae* in the study period, we could estimate the nectar volumes consumed by *Prosoeca* flies. Furthermore, there was no indication of an exchange with surrounding fly populations during the study period [34]. Based on these special circumstances of the study site, we estimated that over a given day, the local fly population was supplied about 0.5 mL of nectar in total, distributed across approximately 400 *Babiana* flowers. Under the assumption that only small amounts of nectar were replaced over the flies’ activity period, we estimated a mean nectar meal size of approximately 1 µL per flower visit. However, additional studies about nectar reproduction and evaporation in *B. vanzijliae* are necessary to give detailed insights on the daily distribution of nectar availability and production rates. The short observation period of this case study was chosen intentionally, in order to give a first estimate on how much nectar a long proboscid fly takes up per flower. An extended study period at this site could possibly have diminished the small population of plants, as nectar measurements require picking the flowers.

### 4.3. Nectar Accessibility

The measured mean nectar volume and sugar concentration of *B. vanzijliae* in the study area was similar to that reported in a previous study about *Babiana* species [21] and matched the general nectar parameters of flowers visited by insects with long sucking mouthparts [52]. The amount of nectar was highly variable even in the morning before the flies were active. This is not surprising since many nectar host plants provide variable amounts of nectar, a strategy that keeps their pollinators “hungry but faithful” and maintains inter-flower movements [46]. We assume that a small and unpredictable amount of nectar per flower is the outcome of a selection process that forces the insects to forage more widely, and therefore, ensuring greater dispersal of pollen. These distinct differences in the nectar reward are in line with previous studies and represents a “bonanza-blank” schedule [53], including both “bonanzas” and “lucky hits”, i.e., high rewarding flowers [54], and “blanks”, i.e., flowers that offer little to no nectar.

Measuring the nectar volume with microcapillaries is a standard field method [51,55,56]; whereas, it is not easy to measure the nectar levels inside the flowers if the flowers are not semitransparent. This study presents a method for calculating the nectar level in floral tubes with known geometry. Using our digital nectar level calculation tool (https://www.geogebra.org/m/se2cwx9s, accessed on 16 April 2021), it is possible to calculate nectar heights from measured nectar volumes and estimate the required proboscis length needed to reach nectar in a given flower tube radius and length. According to the calculations of nectar heights from individual flowers, we can conclude that a *Prosoeca* fly with an average proboscis length could drink at least a small amount of nectar in approximately 95% of the nectar-containing flowers. The flies with minimal proboscis length in the population could reach nectar only in half of the flowers. Longer-than-average flowers, or flowers with lower-than-average nectar quantities, were only accessible to flies with a relatively long proboscis.

## 5. Conclusions

Although there was a one-to-one interaction between *P. marinusi* and *B. vanzijliae* during the study period, it remains uncertain whether *P. marinusi* was the pollinator of its nectar host plant. Since the present field study was not intended for pollination monitoring, we did not investigate pollen transfer or fruit-set. In contrast, we focused on the visiting flies and their morphological adaptations to drink nectar and provided a method for studying nectar accessibility in the local population of flower-visiting insects.

## Figures and Tables

**Table 1:** Body size and proboscis morphometry of *Prosoeca marinusi* (Nemestrinidae) from the population of the study site (Northern Cape Province, South Africa, 31°20′54” S, 19°05′30” E).

*Prosoeca marinusi* Flies	Min–Max [mm]	Mean ± s.d. [mm]
Body length (*n* = 80)	16.21–21.96	19.14 ± 1.25
Proboscis length (*n* = 80)	23.68–38.86	32.63 ± 2.93
Labellum length (*n* = 10) ^1^	0.79–0.89	0.82 ± 0.04
Proximal proboscis (*n* = 10), height ^1^	0.5–0.63	0.65 ± 0.04
Width ^1^	0.31–0.44	0.37 ± 0.04
Distal proboscis (*n* = 10), diameter ^1^	0.24–0,19	0.21 ± 0.02
Labella (*n* = 10), diameter ^1^	0.24–0.18	0.22 ± 0.01

^1^ measured from sections in the lab.

**Table 2:** Perianth tube length and diameter of *Babiana vanzijliae* (Iridaceae) at the study site (Northern Cape Province, South Africa, 31°20′54” S, 19°05′30” E).

*Babiana vanzijliae* Flowers	Min–Max [mm]	Mean ± s.d. [mm]
Perianth tube length (n = 41)	19–38.25	26.92 ± 5.75
Perianth tube external radius (n = 41)	0.75–1.43	0.95 ± 0.12
Perianth tube internal radius (n = 9), mid ^1^	0.37–0.47	0.42 ± 0.03
bottom ^1^	0.28–0.37	0.32 ± 0.04
Stylus radius (n = 9), bottom ^1^	0.22–0.27	0.25 ± 0.02
Floral wall thickness (n = 9) ^1^	0.43–0.60	0.53 ± 0.07

^1^ measured from sections in the lab.

**Table 3:** Measured nectar volumes in flowers of *Babiana vanzijliae* (Iridaceae) before and after flower visits of *Prosoeca marinusi* (Nemestrinidae) and calculated nectar heights inside the perianth tube.

Nectar Per Flower	Volume [µm]Min–Max, Mean ± s.d.	Height [mm] ^2^Min–Max, Mean ± s.d.
Before fly activity (*n* = 44) ^1^	0.1–5.3, 1.54 ± 0.94	0.73–10.37, 5.12 ± 2.63
After flower visit (*n* = 39)	0.0–2.0, 0.52 ± 0.63	0.0–8.87, 2.67 ± 3.09

^1^ Flowers without nectar excluded; ^2^ Calculated with digital GeoGebra tool (https://www.geogebra.org/m/se2cwx9s, accessed on 16 April 2021), see Appendix A.

## Data Availability

The histological sections and the biometrical data are available on request from the corresponding author.

## Appendix A

For the calculation of the nectar height inside the perianth tube of *B. vanzijliae*, we used the disc method known from integration calculus. This method allows for linear functions to be defined to create a two-dimensional model of the tube, which can be rotated around an axis to determine the volume inside the tube. The measurements for the radius of the stylus (*r_s_*), the perianth tube radius at the base (*r_b_*) and at mid-length (*r_m_*), and the height of the tube (*h*) are used (Figure A1) to define two linear functions

(A1) $$f\left(x\right)=\frac{r_{m}-r_{b}}{0.5\times h}\times x+r_{b}$$

representing the wall of the tube, and

(A2) $$g\left(x\right)=r_{s}$$

representing the outer line of the stylus. These functions fit best to the shape of the perianth tube, given that the lower half of the tube contains the nectar. The functions *f* (Formula 1) and *g* (Formula 2) can be rotated around the *x*-axis, creating a three-dimensional figure of the perianth tube (a frustum of a cone) including the stylus (Figure A1).

The volume of the resulting figure is calculated using the disc method.

(A3) $$V\left(x\right)=\pi \times \int_{0}^{h}\left[f{\left(x\right)}^{2}-g{\left(x\right)}^{2}\right]dx=\pi \times \int_{0}^{h}\left[{\left(\frac{r_{m}-r_{b}}{0.5\cdot h}\cdot x+r_{b}\right)}^{2}-r_{s}{}^{2}\right]dx$$

Formula (3) calculates the volume of the whole perianth tube excluding the volume of the stylus, which represents the maximum possible amount of nectar inside the tube. This calculation can be done using the measured values for the radius of the inner diameter of the perianth tube on the bottom (*r_b_*), and at mid length (*r_m_*), the radius of the stylus (*r_s_*), and the tube length (*h*). To calculate the nectar height (*n_h_*) from a given nectar volume (*V*), almost the same formula can be used:

(A4) $$V_{n}\left(x\right)=\pi \cdot \int_{0}^{n_{h}}\left[f{\left(x\right)}^{2}-g{\left(x\right)}^{2}\right]dx=\pi \cdot \int_{0}^{n_{h}}\left[{\left(\frac{r_{m}-r_{b}}{0.5\cdot h}\cdot x+r_{b}\right)}^{2}-r_{s}{}^{2}\right]dx$$

Formula (4) shows the relationship between the nectar volume (*V_n_*) and the nectar height (*n_h_*) inside the perianth tube. Since the nectar volume is known from the measurements, only n_h_ remains unknown in this Equation (4). This equation can then be solved to determine n_h_ (e.g., by using GeoGebra or another mathematical software program).
